# 

**Published:** 1843-12

**Authors:** 


### CONTENTS

OF NO. VI.

### ORIGINAL COMMUNICATIONS.

### ESSAYS AND CASES.

Art. I.—Observations on the Pathology and Treatment of Neuralgia. Communicated to the Medical Society of Columbia, Tennessee. By Thomas Barbour, M.D. 401

Art. II.—Cases of Remitting Fever, with Remarks. By John Dawson. - - - - - - - 418

### REVIEWS.

Art. III.—Physiology Vindicated, in a Critique on Liebig's Animal Chemistry. By Charles Caldwell, M.D. Jeffersonville, Ia: A. Spaulding Tilden, 1843. pp. 95, 8vo. - - - - - - - 430

### SELECTIONS FROM AMERICAN AND FOREIGN JOURNALS.

Sir Astley Cooper. - - - - - - - 462
Sale of Secret Remedies Abroad. - - - - 467
Worari Poison and its Effects. - - - - 468
Dislocation of the External Semilunar Cartilage. - - 470

Anatomy and Physiology of the Spinal Accessory Nerve. - 471
One Advantage of Marriage. - - - - - - 471
Diagnosis between Epididymitis and Orchitis. - - 471

ORIGINAL INTELLIGENCE.

The Western Journal. - - - - - 473
Thou Atomy Thou! - - - - - - 476
Galt's Practical Medicine. - - - - - 476
Pereira's Materia Medica. - - - - - 477
Medical Institute. - - - - - - 477
Index. - - - - - - - 478

